# From marginal to essential: the golden thread between nutrient sensing, medium composition and *Plasmodium vivax* maturation in in vitro culture

**DOI:** 10.1186/s12936-019-2949-x

**Published:** 2019-10-10

**Authors:** Richard Thomson-Luque, John H. Adams, Clemens H. M. Kocken, Erica M. Pasini

**Affiliations:** 1 Center for Infectious Diseases-Parasitology, Heidelberg University Hospital, Im Neuenheimer Feld 324, 69120 Heidelberg, Germany; 2 Center for Global Health, & Infectious Diseases Research, Department of Global Health, College of Public Health, University of South Florida, 3720 Spectrum Blvd, Suite 404 IDRB, Tampa, FL USA; 3 Department of Parasitology, Biomedical Primate Research Centre, Lange Kleiweg, 161, 2288 GJ Rijswijk, The Netherlands

**Keywords:** Malaria, *Plasmodium vivax*, Medium, Continuous long-term blood-stage culture

## Abstract

Historically neglected, due to its biological peculiarities, the absence of a continuous long-term in vitro blood stage culture system and a propensity towards high morbidity rather than mortality, *Plasmodium vivax* was put back on the agenda during the last decade by the paradigm shift in the fight against malaria from malaria control to malaria eradication. While the incidence of the deadliest form of malaria, *Plasmodium falciparum* malaria, has declined since this paradigm shift took hold, the prospects of eradication are now threatened by the increase in the incidence of other human malaria parasite species. *Plasmodium vivax* is geographically the most widely distributed human malaria parasite, characterized by millions of clinical cases every year and responsible for a massive economic burden. The urgent need to tackle the unique biological challenges posed by this parasite led to renewed efforts aimed at establishing a continuous, long-term in vitro *P. vivax* blood stage culture. Based on recent discoveries on the role of nutrient sensing in *Plasmodium*’s pathophysiology, this review article critically assesses the extensive body of literature concerning *Plasmodium* culture conditions with a specific focus on culture media used in attempts to culture different *Plasmodium* spp. Hereby, the effect of specific media components on the parasite’s in vitro fitness and the maturation of the parasite’s host cell, the reticulocyte, is analysed. Challenging the wide-held belief that it is sufficient to find the right parasite isolate and give it the right type of cells to invade for *P. vivax* to grow in vitro, this review contends that a healthy side-by-side maturation of both the parasite and its host cell, the reticulocyte, is necessary in the adaptation of *P. vivax* to in vitro growth and argues that culture conditions and the media in particular play an essential role in this maturation process.

## Background

Overall 3.2 billion people in 95 countries remain at risk of malaria infection [1]. Malaria particularly affects the poorest populations living in the tropical and sub-tropical areas of the world. Deaths are a consequence of a series of complications termed severe malaria and of treatment failure. The high mortality combined with an even higher burden of disease, known as morbidity, is hampering the socio-economic progress of developing countries [2]. In 2007, the Gates Malaria Forum launched a paradigm shift in the fight against malaria moving the discussion from malaria control to malaria eradication. The World Health Organization (WHO) supported the paradigm shift and over the years malaria eradication strategies became an integral part of the traditional malaria control programmes. Six years later, epidemiological data started to emerge suggesting that while successes in the fight against *Plasmodium falciparum* resulted in a decline in the incidence of *P. falciparum* malaria, this notable accomplishment went hand in hand with an increase in the incidence of other human malarias, thus threatening the prospects of eradication [3]. Eleven years later, malaria eradication is still work in progress and is further complicated by the emergence of new human malaria parasites, such as the zoonotic *Plasmodium knowlesi* [4], *Plasmodium simium* [5] and two different species of *Plasmodium ovale* (*P. ovale wallikeri* and *P. ovale curtisi*) [6]. Taken together these findings have led to a growing awareness that the success of malaria eradication programmes [7] hinges on the possibility to tackle all recognized human malaria parasite species: *P. falciparum*, which is responsible for millions of deaths, mainly in sub-Saharan Africa; *Plasmodium vivax*, the geographically most widely distributed human malaria parasite [8], characterized by millions of clinical cases every year, a very high level of morbidity and a widespread spectrum of illness (including severe disease and death); *P. ovale* which, like *P. vivax*, gives rise to asymptomatic hypnozoites infections, *Plasmodium malariae* and the zoonotic *P. knowlesi*. Until recently, much research has focused on addressing the major public health challenge represented by *P. falciparum,* while *P. vivax* and other human malaria parasites have historically been neglected. Due to the number of unique challenges posed by *P. vivax,* such as (1) the formation of sexual transmissible stages called gametocytes from the very beginning of the sometimes still asymptomatic blood stage infection [9], (2) the formation of dormant stages (hypnozoites [10, 11]) in the liver that unpredictably reactivate in a strain-dependent manner [12, 13] to produce relapses and (3) the fact that in terms of invasion it is a reticulocyte-restricted parasite [14], it is especially difficult to interrupt its transmission, to find suitable prevention/treatment strategies and to make progress towards a complete understanding of its biology. This review will focus specifically on the second most important human malaria parasite *P. vivax* and on the major hurdle that has hampered research into this parasite for so long: the impossibility to establish continuous, long-term *P. vivax* in vitro blood stage cultures.

## Malaria blood stage cultures

At present only two malaria parasites, *P. falciparum* and *P. knowlesi* can be maintained long-term in continuous blood stage culture in human red blood cells (RBCs). *Plasmodium falciparum* has been the first parasite to be cultured in vitro [15], while *P. knowlesi* was first adapted to culture in rhesus macaque RBCs [16], and then in human RBCs [17]. The establishment of *P. falciparum* and *P. knowlesi* blood stage culture systems has opened a number of different research avenues into these parasites’ biology and pathophysiology as it has provided scientists around the world with plenty of parasite material for various types of experimentation including reverse [18] and forward [19] genetics, cell biology and most recently ‘omics [20]. Considering the way in which the establishment of blood stage cultures increases the research ability of the scientific community into a specific malaria parasite, the devastating impact on *P. vivax* research of more than 100 years [21] of failed attempts to adapt *P. vivax* to continuous, long-term in vitro blood stage culture growth becomes clear. One of the major stumbling blocks has been the inability to achieve *P. vivax* exponential growth rates between cycles, which are readily observed in in vitro cultures of *P. falciparum* and *P. knowlesi*. In 1997, Golenda et al. [22] reported exponential growth rates for the *P. vivax* Chesson strain cultured in vitro in medium supplemented with human haemochromatosis reticulocytes, but despite repeated attempts to reproduce these results, exponential growth rates for *P. vivax* in human reticulocytes have not been observed since. Typically, *Plasmodium* parasites are cultured in enriched media composed of a water-based solution supplemented with red blood cells/reticulocytes, serum and an antibiotic. Parasite growth in these conditions is a product of three main variables: the ability for host cell invasion, maturation within the invaded host cell and parasite egress and re-invasion into new host cells. An in depth understanding of ways to support each of these three parameters is necessary to address the parasites’ exponential decrease (often until complete disappearance) after it is subjected to in vitro conditions, which has represented the major hurdle to the establishment of long-term in vitro *P. vivax* blood stage cultures.

## Addressing the major hurdle in the establishment of long-term, continuous *Plasmodium vivax* in vitro blood stage cultures

When carefully going through the long history of *P. vivax* culture attempts, there are two major issues, which affect the parasite’s in vitro growth rate, that need to be addressed in order to overcome the exponential decrease of *P. vivax* in vitro.

The first major issue is related to the relative inefficiency, which may in part be due to the lack of inefficiency of the culture system in itself, with which *P. vivax* merozoites in vitro are invading their well-recognized target cell, the reticulocyte [23]. This has two sides to it as it depends both on (i) the ability of schizonts to produce healthy merozoites and their ability to invade their target cells after egress; and (ii) on merozoites being supplied the right target cells for invasion. The question of what the “right target cell” for invasion by *P. vivax* merozoites actually is, has been a hot subject of research and debate. It is now better understood that the cell conventionally called “reticulocyte” is actually a heterogeneous cell population comprising blood cells from different ages [24], where the abundance of specific sub-populations can be characterized by FACS analysis and recently mass cytometry [25], based on the abundance of different surface receptors. It has been shown that some *P. vivax* strains [26], and the murine malaria parasite *Plasmodium yoelii* [27] prefer invading the pool of particularly young cells within the reticulocyte population, as Mons had already suggested in the 1990s [28]. Supporting this are the recent suggestions that parasite alternative ligands EBPII [29], RBP2b [30], RBP1a [31], AMA1 [32], MSP1 [33], GAMA [34], RON2, RON 4 [35] and RON5 [36], acting as first-touch molecules, may be responsible for *P. vivax* specific tropism for reticulocytes and specifically for those expressing higher amounts of the transferrin receptor (CD71), which has itself been proposed as a potential receptor for RBP2b based on expression knockdown analysis, invasion inhibition using PvRBP2b monoclonal antibodies and Cryo-EM structure analysis [30, 37]. It is, however, unclear whether this preference for particularly the younger subpopulation of reticulocytes within the whole reticulocyte population is a general characteristic of all *P. vivax* strains and what role the reticulocyte origin in terms of host (human vs. different new world monkey species) has in the parasite’s tropism [38]. Several factors potentially influence the composition of the reticulocyte population and it is not fully understood how they impact *P. vivax* merozoite’s ability to invade the host cell. Such factors include: (i) the source of reticulocytes utilized (peripheral blood (PB) from healthy donors, PB from haemochromatosis patients, cord blood, haematopoietic stem cell (HSC) cultures or vivax malaria patient’s own reticulocytes [39]), (ii) the methods used for reticulocyte isolation-enrichment (gradients, ultracentrifugation, aqueous multiphase systems of polymers [40] or immune-magnetic sorting [38]) and (iii) the reticulocyte storage conditions (sometimes even for a month at 4 °C [41]). Yet, it seems reasonable to assume that the fresher the reticulocytes and the least they have been subjected to manipulation prior to use, the more likely it is that specific characteristics needed for *P. vivax* invasion are preserved. Nonetheless, it has been proven that frozen HSC-derived reticulocytes can be invaded by *P. vivax* [42]. However, a detailed analysis of reticulocyte surface markers lost over time during maturation, side by side in vivo and in vitro as well as under storage conditions is still needed. Therefore, given the number of variables and the limited knowledge of reticulocytes and their maturation, it is paramount to foster studies in these areas and to promote the collaboration of malaria scientists with red blood cells specialists. However, the study of the target cell is but one side of the story.

**Fig. 1:**
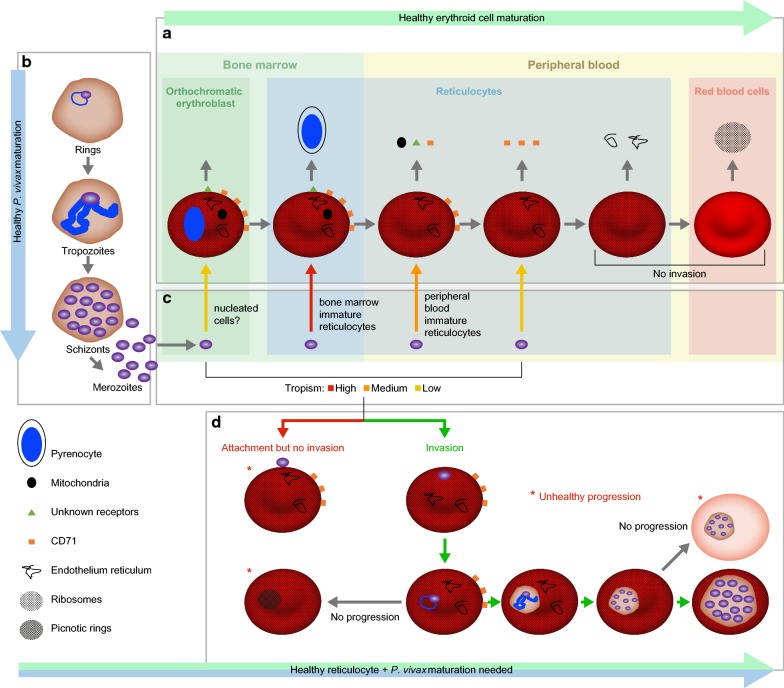
Diagram of the processes occurring during *P. vivax* long-term in vitro blood stage culture. Depiction of processes involved in the development of the parasite (invasion, maturation and egress) and the reticulocytes (maturation) including healthy progression and road-blocks observed in *P. vivax* in vitro long-term culture attempts. **a** The healthy erythroid cell maturation from orthochromatic erythroblast in the bone marrow to mature red blood cell is depicted. During this process the erythroid cell first expels the nucleus in form of pyrenocyte. Thereafter, it loses the mitochondria, different surface proteins (including the CD71) and, finally, the reticulum and the ribosomes. **b** The healthy maturation of *P. vivax* is depicted from rings to trophozoites to schizonts and the egress of merozoites. **c** The tropism of *P. vivax* merozoites for the different cell-types produced during the healthy erythroid maturation is examined and depicted using different arrow colors for high, medium, low. **d** The joint development of *P. vivax* parasite and the reticulocytes they have invaded is portrayed including anomalies in *P. vivax* invasion (adhesion but no invasion) and development (picnotic rings, lack of proper maturation of schizonts), which result in no progression and are marked by a red star

**Fig. 2:**
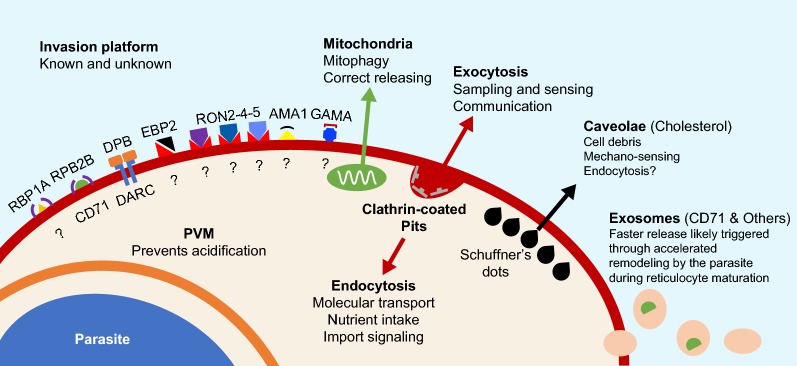
Natural and *P. vivax*-driven reticulocyte remodelling of infected reticulocytes in vitro. Proposed host structures that need to be correctly formed at the *P. vivax*-reticulocyte membrane interface for a healthy side-by-side development of parasite and host cell within the in vitro culture

The second major issue, which hinders *P. vivax* exponential in vitro growth and thus the establishment of a long-term, continuous, blood-stage in vitro culture, is the lack of maturation [36] across the different stages of the life cycle (from rings to schizonts to merozoites). Again, the limited number of parasites able to make their way to the second cycle in vitro cannot be readily attributed to a single cause, as a very complex set of variables is likely to contribute to the scarce parasite fitness in vitro. In this context, particular attention should be given to our current inability to support the parallel healthy maturation of both the parasite and the invaded host cell, which is indispensable in providing the parasite with the right environment for growth and for egress (Fig. 1). During the maturation of a reticulocyte into a RBC, its intracellular and membrane composition constantly changes and so does the parasite, which is involved in its own maturation cycle (Fig. 2). Moreover, it is well known that the parasite establishes “contact” with its blood host cell and extensively remodels it giving rise to a complex system of membranes (parasitophorous vacuole, Schüffner’s dots etc.). It is thus likely that the changes in terms of nutrients and surface membrane organization that accompany the healthy maturation of reticulocytes will affect the healthy maturation of the parasite and its ability to egress at the end of its intraerythrocytic cycle. In this context it has been suggested that an acceleration in the maturation of the host cell may be triggered by *P. vivax* very early post-invasion [26]. However, this in vitro observation needs to be explored further and confirmed independently as this acceleration has not been observed in the *P. vivax* Sal-1 strain in the *Aotus* non-human primate model [38]. Many have suggested that the requirements for *P. vivax* in vitro growth may be more complex and different from those of *P. falciparum.* Brockelman’s [40] repeated but failed attempts at culturing 20 isolates of *P. vivax* using the system described for *P. falciparum* by Trager and Jensen in 1976 [15] strongly support this hypothesis. Parasite fitness (its ability to propagate) in vitro is most likely related to parasite intrinsic factors, to culture conditions as well as, as explained above, to reticulocyte fitness.

## Parasite-intrinsic factors

Numerous strains of *P. vivax*, distinguishable chiefly by their biological characteristics, are known to exist. Two main varieties are recognized: the so-called temperate and tropical strains [43]. It is clear that different *P. vivax* strains and field isolates may exhibit different intrinsic characteristics with regards to their adaptability to long-term in vitro blood stage culture conditions. Even *P. falciparum* shows strain-dependent distinct mechanisms for red blood cell invasion [44–46], which, among other variables, affects their different growth rate. In this context, one clear drawback of field isolates is the limited possibility to reproduce experiments and findings with the same, well-characterized parasite. If the variables affecting the establishment of a long-term, continuous *P. vivax* culture system are to be assessed in a structured way, rather than by serendipitous trial and error approaches, working with well-characterized *P. vivax* strains is paramount. Conversely, not much is known about the intrinsic parasite factors influencing adaptability to in vitro culture, broadening the number of *P. vivax* parasites (including both strains and isolates) with which attempts are made may be key to success as choosing the wrong strain may make it impossible to ever establish long-term, continuous in vitro blood stage cultures. Thus, culture adaptation attempts should be carried out in a controlled environment using well characterized strains and inform attempts to adapt the broadest spectrum possible of *P. vivax* parasites including both field isolates and established strains to long-term, continuous in vitro blood stage culture. Moreover, details of the approaches aimed at assessing the relative importance of different in vitro culture variables on *P. vivax* invasion, growth and egress should be shared in an online platform so that advances can be reproduced and failures are not repeated.

When sourcing *P. vivax* parasites from the field, there are a number of variables linked to the character of a patient’s infection and parasite collection, storage and handling that may influence the parasite’s intrinsic fitness. Such variables should be taken into account and documented in order to provide full experimental disclosure.

Variables related to the *P. vivax* infection status of patients revolve around a patient’s condition when blood is drawn, the moment during the clinical course of infection in which blood is taken and individual immunity to infection. *Plasmodium* spp. are capable of surviving host fever responses but the underneath mechanism for this resistance is still largely unknown [47]. Moreover, the effect of unreported self-treatment prior to blood collection (venipuncture) on *P. vivax* fitness is even less clear. Parasites derived from primary *P. vivax* infections as opposed to relapses as well as parasites obtained from semi-immune patients that have been exposed to multiple *P. vivax* infections may also differ in their intrinsic fitness. Going through the spleen’s interstitial slits while pitting [48] may also have an impact on parasite fitness, although *P. vivax* plasticity is widely accepted, especially in their more mature forms [49]. *Plasmodium vivax* parasites may also differ in their intrinsic commitment to sexual development [50], where a parasite’s production of sexual stages is counterproductive if initial exponential growth in in vitro culture is to be achieved.

Variables related to sample collection, handling and storage techniques [51], can also negatively affect parasite viability. However, the reports currently available on the effects of these variables are often contradictory or insufficiently detailed to allow for informed decision. There is as of yet no standard regarding the most suitable blood collection tubes; lithium heparin, sodium citrate, CPD, ACD have all been used; yet, it has been recommended to avoid EDTA tubes [52] and lithium heparin as they block merozoite invasion by masking their apical surface [53, 54]. The time that a sample can spend at room temperature before been processed or the storage temperatures to be used when a blood sample is transported from a remote out-clinic collection centre to the processing center (37 °C vs RT vs 4 °C) are also other aspects to take into account.

The way in which parasite samples are manipulated in preparation for in vitro culture has also been controversial with some researchers arguing for leukodepletion methods [55] to be implemented, other arguing for it to be avoided and with no agreement among the former on the type of method to be used (cellulose-CF11 columns, non-woven fabric filter [56] or plasmodipur filters [57]). Fresh parasites are generally considered more viable than frozen isolates [58] although it has been reported that [40] the cryopreservation of field isolates even allows viability or parasite transmission from in vitro cultures to mosquitoes [59]. In this context, different freeze-thawing solutions have been explored [60], but insufficient comparisons have been performed to achieve robust conclusions on the best methodology to be used to ensure parasite viability when it comes to frozen isolates.

## Culture conditions

Aside from the factors intrinsic to the parasite discussed above, culture conditions play a major role in the ability of a parasite to adapt to long-term in vitro growth. In the specific context of *P. vivax*, culture conditions do not only have to support parasite maturation, but also proper reticulocyte development as it is likely that the maturation of *P. vivax* and its host cell are intertwined. In fact, defects in reticulocyte maturation and autophagy (e.g. the reticulocyte exosomal pathway) [61] have been found to be at the basis of RBC conditions (e.g. HbE/β-thalassaemia, sickle cells anaemia) that are known to create a hostile environment for parasite intraerythrocytic development [62, 63], thus providing some measure of protection from malaria to carriers. Typically, *Plasmodium* parasites are cultured in enriched media composed of a water-based solution supplemented with host cells (erythrocytes/reticulocytes), human serum and an antibiotic. Procedural culture variables include shaking versus static conditions [22, 38], temperature, initial parasitaemia, starting haematocrit, gassing utensils (candle jar versus hypoxic chambers) and gas composition. During past attempts aimed at establishing a long term, continuous in vitro blood stage culture system for *P. vivax*, these variables have been increasingly scrutinized [38, 64], but no agreement was found on their respective merits and no standardization of procedural variable has been possible. Zhou et al. [65] carried out a study in which they compared *P. vivax* growth in static conditions under different environmental oxygen tensions. They found that low oxygen tension (of between 5 and 10%) best supported *P. vivax* growth in two isolates. However, they also noticed a marked difference between the behaviour of the two isolates under the same in vitro conditions, leading them to conclude that the selection of the *P. vivax* isolate, as mentioned above, may be paramount for achieving long-term in vitro culture. In this context, it is important to consider that gassing conditions may have implications for the choice of medium as different gassing conditions may require media with different buffering capacities. It is generally accepted that the complex set of variables known by the cumulative term “culture conditions” have a serious impact on *P. vivax* maturation in in vitro conditions. Antibiotics (e.g. gentamycin or Pen/Strep), which are routinely added to the medium of in vitro parasite cultures to prevent contamination are known to negatively influence the adaptation of some *Plasmodium* field isolates and strains [66] to in vitro culture, but the influence of specific antibiotics and concentrations on different parasite species, strains and isolates has not been systematically investigated.

**Fig. 3:**
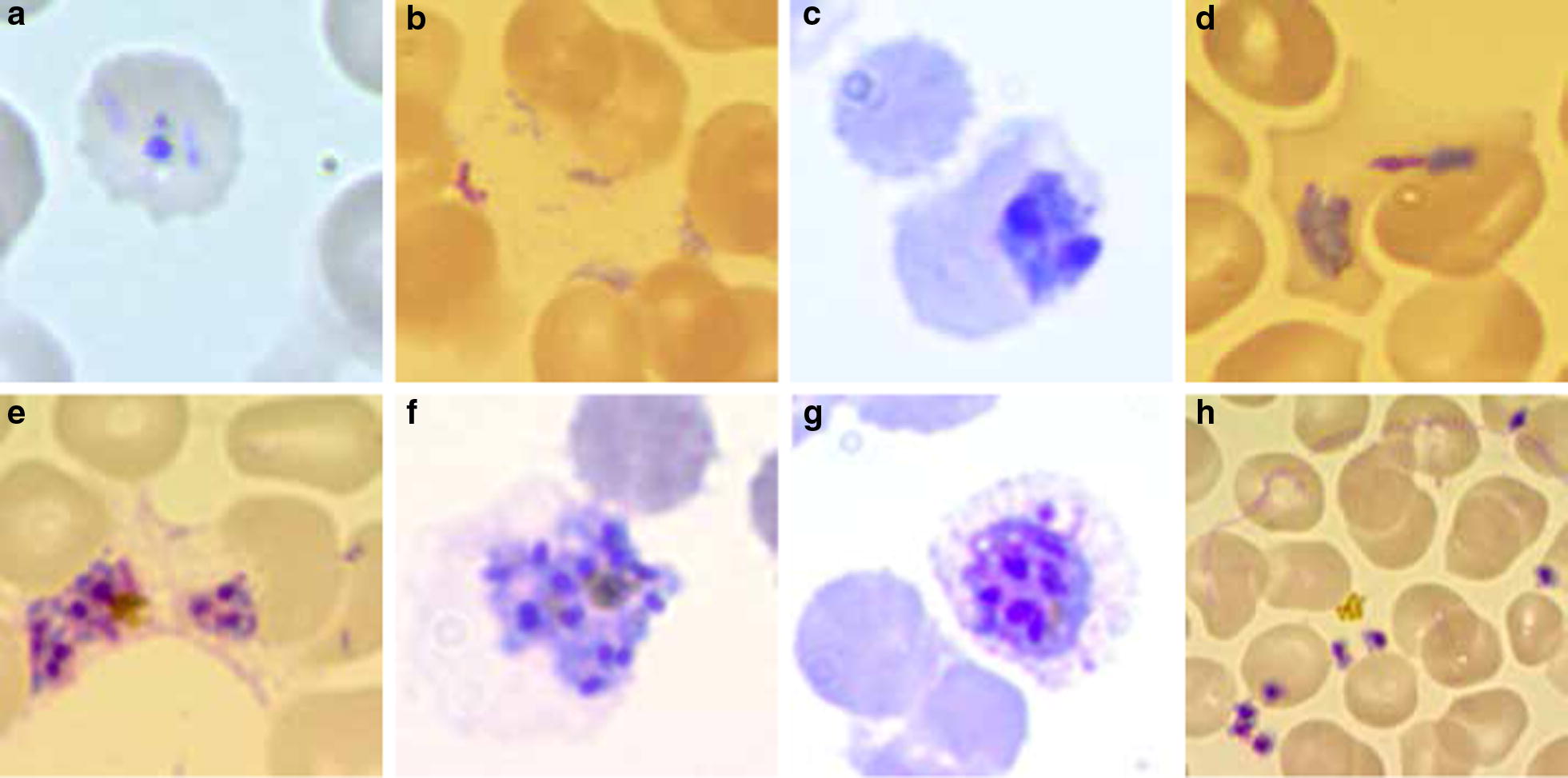
Morfological events characteristics of *P. vivax* in vitro culture failure (images: RTL). **a** Non progressive rings (picnotic), **b** altered trophozoite formation, **c** inhability to replicate, **d** unpacking trophozoite, **e** schizont dismemberment, **f** lack of egress, **g** reticulocyte membrane desintegration and **h** no merozoite invasión

Whether the set of causes responsible for the lack of *P. vivax* maturation (Fig. 3) are to be found in a undetected condition harmful to the parasite that is constantly present in its in vitro culture (e.g. the accumulation of a toxic by-product of metabolism or reticulocyte-derived microparticles [67], which is normally removed by the blood stream or the immediate *P. vivax* environment in vivo) or—in contrast—in a specific factor required by the parasite, which is constantly omitted in the *P. vivax* culture conditions hereto assessed, still needs to be investigated. Overall, the idea of a missing factor has historically spurred researchers to test many different media constituents in search for key players able to promote parasite growth. However, seldom in such research were the needs of the maturing host cell considered. As the scientific community attempts to solve the mystery behind the drowning of exhausted parasites in a hostile environment, the possibility of dormancy [68] within an unhealthy culture environment also needs to be considered.

## Reticulocyte fitness

Reticulocytes are the direct precursors of RBCs and are best understood as an inhomogeneous population comprising very young cells high in CD71, still bearing surface markers such as CD44 as well as old cells that have already lost the CD71 [24]. Due to their continuous maturation, the makeup of this inhomogeneous population is continuously evolving over time. Young reticulocytes undergo a significant re-arrangement in their surface membrane, including the loss of surface markers through vesicle budding and the loss of internal organelles [25]. Towards the end of maturation, the re-organization of surface and cytoskeletal proteins as well as the lipid bilayers will give rise to the biconcave shape characteristic of mature RBCs. Certain *P. vivax* field isolates have shown a specific tropism for young reticulocytes [26], meaning that the dramatic re-modelling that the reticulocytes undergoes during maturation will be even more dramatic as it will occur against the backdrop of parasite invasion. The young reticulocyte thus faces both maturation-related remodelling and parasite-related remodelling. In order to allow for parasite maturation and egress, it is therefore essential that all these remodelling events be coordinated in such a way as to allow for the healthy maturation of the reticulocytes. To this end, the medium will need to contain the appropriate balance of nutrients to allow for the reticulocytes’ membrane evolution and organelle loss to take place alongside the parasites’ segmentation to invasive merozoites. While with a mature population of homogenous RBCs, this seems relatively easy to achieve in vitro, in the case of inhomogeneous reticulocyte populations it may be more difficult and part and parcel of the difficulties of adapting *P. vivax* to long-term in vitro growth.

Hereafter, a closer look at the different media components is taken that have historically been used in various *P. vivax* long-term blood stage culture attempts and try to analyse their pros and contras and the reason why they were selected. Finally, choosing the best of every media may lead to a new *P. vivax* in vitro culture composition that can be tested in the field in the future.

## Culture media tested in *P. vivax* in vitro culture attempts up to date

It is generally accepted that a water-based culture medium containing various micronutrients (e.g. salts) and able to provide a source of carbon (e.g. glucose), amino acids and nitrogen will support *P. vivax* in vitro survival. Except for early reports [21] describing the use of Ringer’s lactate solution (Locke’s fluid) as a medium for *P. vivax* in vitro culture and some extravagant media based on coconut milk [69], initial attempts in the early 70s and 80s mirrored successful *P. falciparum* culture conditions. In most of such attempts the nutritionally complex medium RPMI-1640 was used, but alternative commercially available media have also been tested alone or in combination such as Waymouth, the customized SCMI 612 medium developed by Mahidol University and the most popular medium used for *P. vivax* in vitro culture nowadays, McCoy 5A (Table 1).

**Table 1:**
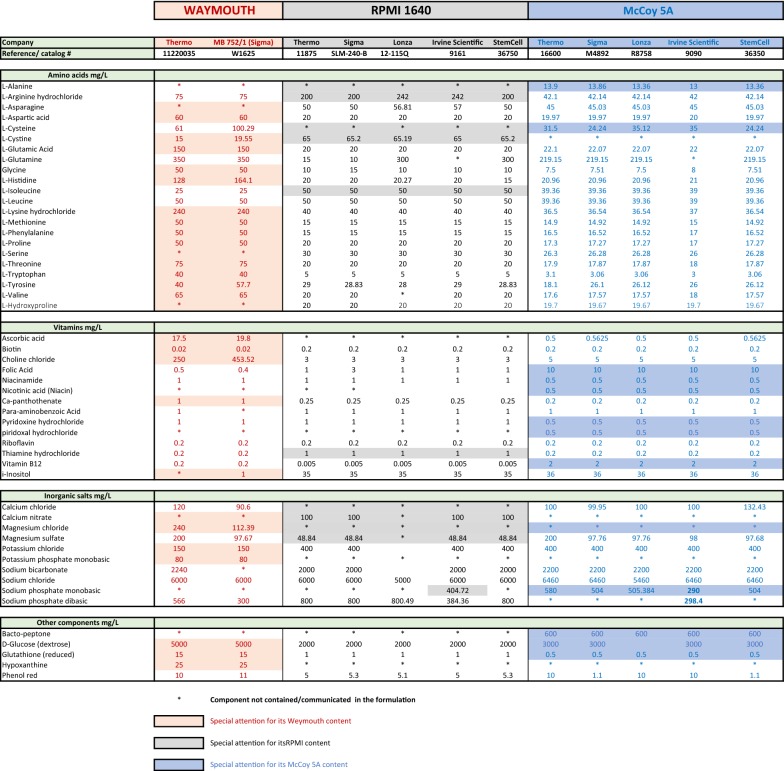
Comparison of the components of the 3 historically most-used media in attempts aimed at culturing *P. vivax* in vitro

Media composition of RPMI-1640, Waymouth and McCoy5A from commonly available commercial brands. Shadowed are components of special interest. *Waymouth* medium is available either powder or liquid and it is highly enriched in amino-acids, ascorbic acid and glutathione. With a higher concentration in glucose than most other commercial media. It is also the only medium used for cultivating *P. vivax* that includes hypoxanthine in its formulation. *RPMI*-*1640* can be sourced in either its powder or liquid forms from a number of different companies. Modified versions of this medium are also commercially available: in such versions the RPMI-1640 is typically supplemented with or depleted of l-glutamine, sodium bicarbonate, HEPES (15 mM, 20 mM or 25 mM), folic acid, methionine, cysteine, Glutamax as well as different amounts of glucose. Special formulations known as the ATCC modification (containing high glucose, low sodium bicarbonate, sodium pyruvate, HEPES, and low l-glutamine) or the Dutch modification (addition of HEPES, with lower sodium bicarbonate (1 g/L)) are also obtainable. *McCoy’s 5A*, unlike other media, contains bacto-peptone, and high levels of glucose. Moreover, it contains high levels of folic acid and thiamine while the levels of cysteine and leucine are reduced compared to other media formulations. McCoy5A contains no magnesium chloride and lower levels of glutathione compared to RPMI and Waymouth. Last, McCoy5A comprises an increased concentration of pABA, and folic acid compared to different commercially available RPMI and Waymouth media. Commercially available in powder and liquid forms too, it includes variant formulations comprising the addition of HEPES, Glutamax, l-glutamine and extra sodium bicarbonate

The RPMI-1640 formulation was first published in 1967 by Moore et al. [70] from the Roswell Park Memorial Institute, where RPMI is the acronym for the name of the institute. It is based on a relatively high concentration of phosphate and is formulated for use in a 5–10% CO_2_ atmosphere. The RPMI-1640 has traditionally been used for the serum-free expansion of normal and neoplastic lymphoid cells as well as bone marrow, hybridomas, HeLa, Jurkat, MCF-7, PC12, astrocytes, and carcinoma cells. With a bicarbonate buffering system (2.0 g/L), in its typical basic formulation (pH = 8), it differs from most mammalian cell culture media.

Long-term *P. falciparum* and *P. knowlesi* [17, 71] in vitro cultures have been achieved using RPMI-1640. However, RPMI-1640 only supported short-term in vitro cultures of the human parasite *P. malariae* [72, 73] the simian malaria parasites *Plasmodium fragile* [74], *Plasmodium cynomolgi* [75], *Plasmodium gonderi* [76] and *Plasmodium inui* [77], and the rodent malaria *Plasmodium berghei* [78]. Among the avian malarias, short-term attempts to cultivate the 12A strain of *Plasmodium lophurae* extracellularly [79, 80] also relied on RPMI and RCE supplemented with duck erythrocyte extracts and thus rich in potassium [81]. However, it is clear that RPMI-1640 has serious limitations when it comes to supporting the long-term in vitro growth of *Plasmodium* parasites other than *P. falciparum* and *P. knowlesi*. In the case of the rodent malaria, *Plasmodium chabaudi* [82], RPMI-1640 allows the parasite to survive, but a combination of three-parts BME Basal Medium Modified with one-part William’s Medium E (Additional file 1: Table S1), better supported both invasion and the rate of maturation of newly invaded rings to young trophozoites as assessed by radioisotope incorporation by *P. chabaudi* trophozoites. Nonetheless, even this media combination did not support exponential parasite growth, thus making it impossible to establish long-term in vitro *P. chabaudi* blood stage cultures.

In terms of attempts at establishing a long-term, continuous *P. vivax* blood stage culture, in the 1970s [83] and 1980s [84, 85] RPMI-1640 was already the medium of choice. It was used first by Mons et al. [86] to study *P. vivax* growth and reinvasion of *Aotus nancymai*’s RBCs in vitro and by Barnwell et al. [87] for the evaluation of the role of the Duffy blood group in *P. vivax* merozoite invasion of erythrocytes. Around the same time, Brockelman et al. [88] started to investigate the parasite’s requirement in terms of metal ions and vitamins and found that a 2:1 mixture of RPMI:Waymouth supported both *P. vivax* nuclear growth (Waymouth) and cytoplasmic differentiation (RPMI). However, attempts at medium optimization by Brockelman et al. ultimately failed to support exponential *P. vivax* in vitro growth. Further attempts to supplement RPMI were carried out in the 90s by addition of liver extracts [89].

Trager’s continuous-flow method to culture *P. vivax* used by Lanners [90] also relied on RPMI medium. Attempts at culturing *P. vivax* using RPMI by Basco et al. [91], Pazarbas et al. [92], Usha Devi et al. [93], Chotivanich et al. [94], and Tasanor et al. [95], were the last reported before a general tendency emerged towards replacing RPMI with McCoy 5A as the preferred medium for culturing *P. vivax* parasites. However, RPMI has recently been used in the short-term in vitro culture of *P. vivax* field isolates using umbilical cord blood by Udomsangpetch et al. [96] and for tests aimed at enhancing ex vivo intra-erythrocytic enrichment and maturation of *P. vivax* for rapid sensitive parasite growth assays [97].

Waymouth medium MB 752/1 was originally developed by Charity Waymouth at the Jackson Laboratory in Maine, USA [98]. It consists of a synthetic medium (Table 1) for the cultivation of mouse L929 cells, but its applicability has been extended to include whole organ culture and the establishment and growth of carcinoma cell lines from pleural effusions.

Brockelman and colleagues, in the 1980s [89, 99], were fond of mixing Waymouyh with RPMI, most probably with the intention of taking advantage of each medium’s composition. They observed that while the use of RPMI gave pronounced cytoplasmic differentiation, nuclear growth was handicapped if Waymouth was not added in the RPMI/Waymouth 2:1 ratio mentioned above. More recently, drug sensitivity studies [95] and a series of comparisons aimed at improving culture conditions for long-term in vitro culture of *P. vivax* by Roobsoong et al. [61] have again employed Waymouth.

SCMI 612 was first tested by Brockelman et al. [99] at the Department of Microbiology in the Faculty of Science at Mahidol University, Bangkok, Thailand. SCMI 612 was prepared with a base of 90% balanced salts solution, to which 1% vitamin solution, aminoacids and 1% of a 0.1 mg/mL hypoxanthine solution were added. Brockelman suggested that this medium would work better than either RPMI 1640 or Waymouth media since at 44 h post-maturation, mature schizonts with 12–20 merozites were more frequent in in vitro cultures with SCMI612 medium. No more attempts utilizing SCMI 612 since have been reported in the literature.

In 1959, initially using Basal Medium 5A, Dr. Thomas McCoy modified it for the in vitro cultivation of Novikoff hepatoma cells [100]. Nowadays this medium is known as McCoy’s 5A. Subsequently, Hsu and Kellogg employed this medium to support the growth of primary bone marrow cultures as well as skin, gingiva, testis, mouse kidney, omentum, adrenal glands, lung, spleen, rat embryos, and other tissues [101]. McCoy’s 5A uses a sodium bicarbonate buffer system (2.2 g/L) and is, therefore, also suitable in combination with a 5–10% CO_2_ environment to maintain a physiological pH. Among the commercial media used in attempts aimed at *P. vivax* culture, McCoy5A is the only one including l-alanine in its formulation (Table 1). McCoy’s 5A has been the media most widely used in attempts to culture *P. vivax* in vitro in the last two decades (24, 26, 38, 39, 41, 42, 52, 60, 61, 94). A list of the components of every commercially available RPMI-1640, Waymouth and McCoy5A media can be found in Table 1 where significant differences between the media are highlighted.

Non-commercially available Modified Harvard medium (BGM) was created at Harvard Medical School by Nobel Prize winner Christian B. Anfinsen to define the factors affecting *P. knowlesi* growth by using a medium containing only substances of known structure and purity [102]. It was found that for long-term propagation serum was needed. This synthetic medium demonstrated the need for glucose, pABA and l-methionine. Modified Harvard medium was thereafter used for the culture of *P. lophurae*, *P. cynomolgy*, *P. falciparum* as well as for attempts at culturing *P. vivax* that were summarized by Gieman and McKee as: “*Growth, no significant multiplication*” [103]. Some other avian malaria parasites (*Plasmodium gallinaceum*, *P. lophurae*, and *Plasmodium fallax)* as well as the rodent malaria *P. chabaudi* were cultured short-term in vitro using BGM by Trigg et al. [104]. Noteworthy, is the systematically carried out attempt by Spandorf and Manwell to adapt the avian malaria parasites *Plasmodium circumflexum, Plasmodium hexamerium* and *Plasmodium vaughani* to in vitro blood stage growth [105]. In these attempts, the BGM was used, supplemented with coenzyme A, ATP, thioctic acid and K-malate, alone or in combination. In these studies, *P. hexamerium* parasites increased fourfold in numbers in 72 h and proved to be viable on sub-inoculation. Also, the multiplication of *P. circumflexum* up to eight times their original number was obtained in 48 h when coenzyme A, pork liver extracts and K-malate were added to the medium. The efficient maturation *P. circumflexum* at 72 h in these conditions was also noteworthy. Yet, long-term in vitro culture of these parasites could not be established.

A medium tested in attempts to culture the rodent malaria parasite *P. chabaudi* in the past, and recently been used [97] in attempts at culturing *P. vivax*, is Eagle’s Basal Medium (BME) [82]. There are several “basal” media described by Harry Eagle that vary slightly from one another. BME is one of the most widely used synthetic cell culture media and was developed in the late 1950s with the aim to fulfill essential nutritional requirements and provide all factors critical to growing cells in culture. BME is the predecessor of Eagle’s Minimum Essential Medium (MEM), Glasgow’s Medium and Dulbecco’s Modified Eagle’s Medium (DMEM), which has also recently been tested for *P. vivax* [97]. BME contains eight B vitamins, ten essential amino acids and includes cystine, tyrosine, glutamine, very little biotin, while lacking vitamin B12 and pABA. It is also available with and without Earle’s salts, l-glutamine and sodium bicarbonate. BME, F10, GMEM, M199, MEMalpha, MarrowMax, Stempro 34, OBM, HPGM, Stemline II, Stemspan ACF, Stemspan H3000, DMEM and its variant DMEM/F12, which includes a nutrient mixture containing linoleic acid, lipoic acid, hypoxanthine and putrescine, were all found to be inferior to RPMI-1640 and McCoy 5A in terms of their support of *P. vivax* growth [97]. However, the addition of putrescine to the latter is worth noticing as it has been found to boost the rings transition into trophozoite stage in *P. falciparum* treated with DFMO [99]. The exception to this long list of media failures is the reported survival of half the *P. vivax* parasite during maturation obtained using Iscove’s Modified Dulbecco’s Medium (IMDM) [97]. Although half of the parasites are still lost, results suggests that IMDM may be able to protect intraerythrocytic *P. vivax* from death and haemolysis during its transition from early to late stages.

## Towards a medium for *P. vivax* in vitro culture: a detailed analysis of medium components

### The right pH: sugars and alternatives to current practices

All media used in cultures of *Plasmodium* species *(Plasmodium spp.*) contain a synthetic mixture of inorganic salts as a basis. Among the functions of these inorganic salts are to maintain a proper pH as well as an ideal osmotic pressure and to provide a source of energy for the parasite. When selecting a specific medium, it is important to consider its characteristics in the context of other culture conditions such as e.g. the gas mixture used, which can influence the final pH.

Growth of parasites in a nutritionally complete culture medium is generally optimal when the medium is buffered at a pH in the range of 7.2–7.4. Sodium bicarbonate is the most commonly used buffer in commercial media intended for cultivating *Plasmodium* spp. in vitro [106]. However, sodium bicarbonate has a pKa of 6.3 at 37 °C, resulting in suboptimal buffering throughout the physiological pH range [107]. Under these circumstances there are two approaches to equilibrate the media: (1) the use of Hanks’ balanced salt solution (such as Dr. Hsus recipe for McCoy5A), which theoretically allows its use with air in a closed system at a low concentration of sodium bicarbonate, and (2) the use of the most extended, but more difficult to handle, Earle’s balanced salt solution with higher concentration of sodium bicarbonate but intended to be used with a 5% CO_2_ gas mixture to avoid a pH increase at standard 37–37.5 °C incubation temperature. An alternative method is to use a medium able to produce sufficient buffering capacity even in the absence of a 5% CO_2_ gas mixture. This can be achieved by using a medium containing Earle’s salts and a lower sodium bicarbonate concentration, of about 0.85 g/L. A commercialized RPMI medium (known as Dutch modification) contains lower sodium bicarbonate (1 g/L) while being supplemented with HEPES. This medium has never been used to culture any of the *Plasmodium* spp. A very different approach was devised in 1963 by Leibovitz [108], who used the buffering capacity of free base amino acids and completely omitted sodium bicarbonate from his L15 medium (a comparison of the components of L15 with those of RPMI-1640, Waymouth and McCoy5A is provided in Additional file 1: Table S1). By also adding pyruvate, and substituting galactose for glucose, he obtained a pH of 7.8, which remains very stable, thus making it possible to grow cells in open culture templates. By substituting galactose with 10 mM fructose it is also possible to modulate the pH, and the rate of production of cellular catabolites [109], thus it would be possible to bring the pH in a range thought to be suitable for parasite in vitro growth. However, as glucose was found [88] to be an essential nutrient for the parasite that could not be replaced by ribose, mannose, fructose, galactose, and/or maltose, it is likely that parasite media based on Leibovitz will have to include a mixture of sugars to support parasite growth in their formulation. Furthermore, Brockelman [99] reported that higher glucose levels (3 mg/mL) were needed for *P. vivax* in vitro growth when compared to *P. falciparum.*

Another medium with similar characteristics to Leibovitz’s L15 is SR1-8 [109]. As many other culture media, the Leibovitz and SR1-8 also include phosphates to increase their buffering capacity. Neither of these two media or the modifications thereof have ever been used in attempts at culturing malaria parasites. The most commonly used alternative to bicarbonate is HEPES buffer, which was first described by Good et al. [110]. HEPES acts as a zwitterion and has proven superior in buffering capacity compared to conventional bicarbonate. Thus, it does not require an enriched atmosphere to maintain the correct pH but may be toxic for malaria parasites at concentrations greater than 40 mM. Studies have indicated that 20 mM HEPES is the most satisfactory concentration of the buffer when either Hanks’ and Earle’s solutions are used. CO_2_ incubators should not be used with media buffered solely with HEPES. It is recommended for the sodium bicarbonate concentration not to exceed 10 mM when the HEPES concentration is 20 mM.

### Salts, osmolarity and conductivity: key for achieving correct segmentation

First Trager [111] and then Sherman [79] recognized the importance of salt ions for malarial parasites. Although initially sodium and potassium were the focus of attention, Brockelman demonstrated that enriching RPMI medium with either KCl or NaCl did not boost *P. vivax* growth [99]. Measurements comparing ionic strength of RPMI and Waymouth in terms of specific conductivity have shown that RPMI presented lower conductivity (11.96 mS/cm) compared to Waymouth (13.27 mS/cm) as well as lower osmolarity (296 mmol/kg and 305 mmol/kg respectively). The best results on achieving complete segmentation in schizogony was obtained with an ionic strength of 12.6–12.80 mS/cm when RPMI and Waymouth were mixed at the above-mentioned 2:1 ratio. Through time, the focus of research on inorganic salts shifted to determining the effect of other salts such as magnesium. Magnesium plasma levels ranges between 1.5 and 2.5 mM [112]. Magnesium chloride supports parasite viability when the salt is added to RPMI [24]. When mixing RPMI with Waymouth, the concentration of magnesium increases from 0.45 to 0.92 mM. Yet, the best results in Brockelman’s studies [88] on in vitro schizogony of *P. vivax* were obtained when the mixture of media was enriched with additional magnesium to 1.8 mM, which led to a percentage of ‘segmenters’ (maturing schizonts) as high as 72.61%, highlighting the importance of magnesium supplementation.

Finally, manganese (Mn^2+^) may be essential for *P. vivax* invasion although its importance in enhancing parasite growth and maturation has yet to be definitively proven. Manganese containing buffers have been described as essential for the in vitro re-composition of murine erythroblastic islands [113] after the structure of the bone marrow niches had been altered during bone marrow aspiration. α4/β1 integrin dimers in very immature reticulocytes (the ones that *P. vivax* prefers to invade) can present multiple activation states, the most active of which is reached when divalent cations such as manganese are present in the media [114]. Whether manganese plays an important role for *P. vivax* merozoites binding to and invasion into young reticulocytes still needs to be assessed (Table 2).

**Table 2:** Proposed systematic comparisons needed to unravel key avenues for the development of a continuous in vitro culture system for *P. vivax* blood stages

Media component	Proposed systematic comparisons with current practice	Expected effect on the parasite	Expected effect on the reticulocyte	Media formulation to accomplish proposed action	References
	Earle’s salts + reduce sodium bicarbonate to 0.85 g/L	Optimal buffering capacity: parasite healthy maturation	Reticulocyte healthy maturation	RPMI Dutch’s modification	Never tested
	amino acids + mixture of sugars; no sodium bicarbonate	Optimal buffering capacity: parasite healthy maturation	Reticulocyte healthy maturation	L15 and SR1-8	[106]
	HEPES 20 mM	Optimal buffering capacity: parasite healthy maturation	Reticulocyte healthy maturation		[108]
Inorganic salts	Ionic strength of 12.6–12.80 mS/cm	Better segmentation	Reticulocyte healthy maturation	Waymouth	[86]
	Increase Magnesium chloride 1.8 mM	Increased parasite viability	Appropriate conformation of surface proteins	Waymouth/L15	[86]
	Addition of calcium pantothenate	Enhanced parasite survival and multiplication	If any, unknown	SCMI 612	[40]
	Addition of manganese	Enhanced parasite invasion	Appropriate conformation of surface proteins		[111, 112]
	GlutaMAX	Does not break down to form toxic ammonia: better progression to late stages	Reticulocyte healthy maturation	Commercial McCoy5A/RPMI commercial + glutamax	[35]
	Increase leucine, phenylalanine, valine, isoleucine and methionine	Parasite growth and detoxification of the toxic free heme	Healthy reticulocytes	In most media formulations; concentration may vary	[12, 103, 106, 115–123]
Amino acids	Ascorbic acid	Enhanced parasite survival and multiplication	Healthy reticulocytes: helps to keep proper redox equilibrium	In most media formulations; concentration may vary	[86]
	Orotic acid	Enhances parasite redox stability	Healthy reticulocytes: helps to keep proper redox equilibrium	Needs to be added, concentration to be determined	[59]
Vitamins	pABA concentrations	Enhanced intracellular development and maturation	Healthy reticulocytes	Most appropriate concentration to be determined	[31, 128.130]
	Piridoxyne/pyridoxal hydrocholoride	Enhanced intracellular development and maturation	Healthy reticulocytes	IMDM has highest concentration at 4 g/L	[127, 142, 144]
Serum	Use serum (ideally commercial); serum is likely better than Albumax	Supporting parasite viability	Membrane integrity and maturation	Commercial serum is desirable for inter laboratory standardization	[179–194]
Antibiotics	Gentamicin/other antibiotics	Possible toxicity on specific parasite species and strains	If any, unknown		[64]
Hypoxanthine	Necessary	Parasite maturation	If any, unknown	Waymouth, DMEM/F12	[36]
Lipid	Addition of fatty acids, choline, myo-inositol, ethanolamine, lyso-PC	Parasite survival, maturation and multiplication	Membrane integrity and maturation	Needs to be added, concentration to be determined	[167–172]
Cholesterol	Use lipoproteins containing cholesterol instead of free cholesterol	Parasite maturation and eggress	Membrane integrity and maturation	Needs to be added, concentration to be determined	[175]

Expected effects of proposed approaches on both the parasite and the reticulocyte

### Precursors of DNA/RNA: indispensable for parasite maturation

Purines and pyrimidines are essential for *Plasmodium* parasites development. Neither malaria parasites nor the RBC within which they reside are capable of de novo biosynthesis of purines and, therefore, the parasites must obtain them through salvage pathways [115]. Conversely, whilst erythrocytes lose their capacity to de novo synthesize pyrimidines during maturation, *Plasmodium* spp. are still capable of the de novo biosynthesis of pyrimidine rings. Hypoxanthine acts as a substrate and nitrogen source required for nucleic acid synthesis and energy metabolism and has been widely added to *P. vivax* culture media [36] as it was found to be indispensable for parasite maturation [99].

### Amino acids: GlutaMAX and hydrophobic amino acids

Growth of malaria parasites in media not supplemented with amino acids is markedly reduced. The use of amino acids mixtures for additional supplementation of *Plasmodium* spp. culture media has also been reported [99] and several algal and cell free mixtures are commercially available.

Glutamate, glutamine, cysteine (which can also be replaced by cystine), tyrosine and proline are important as a group of amino acids that are minimally required for parasite growth [108]. Longer persistence of parasites has been found in the cultures supplemented with GlutaMAX, a stabilized form of l-glutamine which does not break down to form toxic ammonia like traditional l-glutamine [38] and, therefore, its use in *P. vivax* in vitro culture attempts is recommended (a list of potential medium improvements vis-à-vis of and alternatives to current practice are suggested in Table 2). In in vitro cultures, human haemoglobin lacks isoleucine and no de novo synthesis of this amino-acid takes place in the parasite; therefore, it must be obtained from the host serum [108]. Isoleucine and methionine have been proven to be of paramount importance for parasite growth [103, 116–124] as together with other hydrophobic amino acids such as leucine, phenylalanine and valine, isoleucine and methionine play a crucial role in the detoxification of the toxic free haem liberated by the digestion of haemoglobin by *Plasmodium* spp. These hydrophobic amino acids namely contribute to its polymerization in a crystalloid form called haemozoin. Altering the process of haemozoin formation undoubtedly or promoting the presence of free radicals leads to parasite death as it has been proven that the anti-malarial drug chloroquine interferes with the formation of haemozoin by capping haemozoin molecules, thus preventing further biocrystallization of haem and leading to haem buildup. Although the levels of these five hydrophobic amino acids in RPMI and McCoy5A are above the normal physiological range [125] in the human body (Table 1), these amino-acids should be considered as of paramount importance for parasite survival and, therefore, an increase in their supply to *P. vivax* through the medium needs to be explored (Table 2).

### Vitamins and related substrates: the need of revisiting the importance of ascorbic acid, orotic acid and PABA through systematic comparisons

The requirement for ascorbic acid by *Plasmodium* spp. has long been a subject of controversy. In monkeys, the deficiency of ascorbic acid was found to inhibit multiplication of *P. knowlesi* [80]. Still, its omission had no in vitro effects [126] when evaluated within the first 24 h of culture. Several groups have succeeded in culturing other simian malaria parasites [17, 71, 76] using RPMI depleted of ascorbic acid. Nevertheless, these data on the need of ascorbic acid by the parasite must be interpreted cautiously as they mostly refer to short-term cultures, may be parasite specific and human serum which was a standard component of the complete medium in these experiments contains ascorbic acid in the range of 0.6 to 1.2 mg%. When tested in the human *Plasmodium* spp. *P. falciparum*, raising the concentration of ascorbic acid in the media above serum level had no significant role on its intraerythrocytic schizogony. Strikingly, *P. falciparum* has been suggested to be able to propagate in a completely defined medium lacking both serum and ascorbic acid [127]. As for *P. vivax,* results of a series of experiments using *P. vivax* field isolates revealed that ascorbic acid is essential to parasite survival and differentiation to segmenters [88]. This series of experiments found higher parasite counts in medium containing at least 6 g/mL ascorbic acid and a parasitaemia decline proportional to the decrease in the concentration of ascorbic acid, the critical point being 3 g/mL. The difference in minimal requirements appeared to be more distinct when the culture time was prolonged to 44 h indicating the importance of ascorbic acid for maturing *P. vivax* schizonts. Thus, its inclusion in all media used in forthcoming efforts at culturing *P. vivax* in vitro is encouraged (Table 2).

Orotic acid is manufactured in the body via a mitochondrial enzyme, dihydroorotate dehydrogenase or a cytoplasmic enzyme of the pyrimidine synthesis pathway. In his studies of nutrients and antimetabolites [128], Jensen observed that orotic acid could not supplement glutamine-deficient medium. Yet, anti-malarial effects of riboflavin antagonists are partially reversed by the addition of orotic acid pointing at dihydroorotic acid dehydrogenase as an important enzyme for *Plasmodium*. Orotic acid (360 μM) was employed by Roobsoong et al. [61] in her attempts to improve culture media conditions for *P. vivax*, but the benefits of adding orotic acid are not discussed. Neither McCoy5A nor RPMI, Waymouth, SCMI 612 or William’s contain orotic acid in their formulations (Table 1; Additional file 1: Table S1).

Observational studies on the antimalarial effect of sulfanilamide in 1940 [129] provided the first evidence for the role of pABA (4-aminobenzoic acid) in the intra-cellular development of *Plasmodium* erythrocytic stages. Indeed, pABA and glucose were found to be fundamental nutritional requirements for the growth of *P. knowlesi* in vitro [102] where pABA appears to be key for the synthesis of folates [127, 130]. In this context, Trager had previously shown that the development of the human malaria parasite *P. falciparum* in human erythrocytes was better when high concentrations of folic acid (1.7 µg/mL) were added to the medium [119]. Sulfonamide-related growth arrest of *P. gallinaceum* was found to be reversed by the addition of pABA [131]. Moreover, parasite growth in both *P. berghei*-infected rats, *P. knowlesi*-infected rhesus macaques and *P. cynomolgi*-infected *Aotus* monkeys was hampered in a diet poor in pABA e.g. milk [128, 129]. But our ultimate understanding of the mechanisms and essentiality of folate metabolism in *Plasmodium* parasites has evolved considerably since the first reports from Trager back in 1958 [121]. Folate de novo synthesis in the parasite [128] is nowadays unquestionable. While in the past media devoid of pABA were common, [132] nowadays, all commercial media contain similar amounts of pABA.

Few other vitamins have been demonstrated to be essential as already suggested by Trager [117]. The role of Vitamin A is controversial [133, 134]. Thiamine (vitamin B1) and riboflavin (vitamin B2) have been found to be essential for the multiplication and growth of different malaria parasites including *P. berghei* [135], avian parasites [136, 137] and *P. knowlesi* [138]. Riboflavin was also found to correlate with infection and severity of malaria in infants [139]. Thus, the ideal medium concentration in vitamins A, B1 and B2 required to foster *P. vivax* in vitro adaptation and growth warrants further investigation. Concentrations of thiamine and riboflavin vary in commercial media; some Waymouth formulations are particularly enriched in these vitamins (Table 1).

Since the 1940s, Trager’s experiments with different *Plasmodium* spp. helped elucidate the importance of calcium pantothenate (Vitamin B5) for parasite survival and multiplication [44, 116–118]. According to Trager [121], pantothenate is not directly used by *Plasmodium* parasites but rather employed as a precursor of coenzyme A (CoA). Trager argues that malarial parasites (or at least *P. lophurae*, the one species with which appropriate studies have been done) have in their erythrocytic stage a major biosynthetic lesion. For their CoA, which is essential for the oxidation of glucose via the citric acid cycle and for the synthesis of cellular constituents of the parasite, they depend completely on the biosynthetic activity of their host erythrocytes. Based on Trager’s findings, Brockelman [99] included calcium pantothenate in his SCMI 612 culture medium for *P. vivax*. Waymouth medium and Williams medium (developed by Williams and Gunn in 1971 for the long-term culture of adult rat liver epithelial cells; formulation in Additional file 1: Table S1) both contain higher concentrations of pantothenate compared to commercially available RPMI and McCoy 5A (Table 1; Additional file 1: Table S1). No attempts using William’s medium for *P. vivax* in vitro culture have been reported.

Piridoxyne (Vitamine B6) has also been studied in connection to *Plasmodium* growth [128]. Historically, *P. vivax* parasites have been known to almost exclusively invade Duffy^+^ populations [140]. However, this paradigm is nowadays under revision as more cases emerge of *P. vivax* infection in Duffy-negative people [141]. Interestingly, pyridoxal kinase activity is known to be decreased in red blood cells of Afro-Americans and it has been postulated that the relatively high frequency of low RBC pyridoxal kinase activity in native Africans may have been selected for by *P. falciparum* malaria [142]. Thus, it is easy to speculate that the right levels of pyridoxine may be an important variable for *P. vivax* intra-erythrocytic development. Biotin (vitamin B7) was the first vitamin said to be involved in the course of *Plasmodium* development. In vivo, malaria parasites as well as the host’s immune system need biotin. Biotin is among the cofactors essential to the early intraerythrocytic development of *P. knowlesi* [125]. Indications, derived from experiments with *P. lophurae and P. cathermerium*, are that it is required at concentrations lower than those at which the host’s immune system, would be adversely affected [119].

### Cellular antioxidants: two redox systems in search for equilibrium

*Plasmodium vivax* malaria parasite infection is known to increase the oxidative stress in the host [143] both locally and systemically through both, *P. vivax*-specific mechanisms (e.g. maximum decline in erythrocytic superoxide dismutase [144], and glutathione peroxidase activities [145] accompanied by pronounced iNOS expression [146]) and non-specific mechanisms (e.g. increase in malondialdehyde [147, 148]). Beside playing an important role in the pathogenesis of *P. vivax*, the antioxidant equilibrium undoubtedly plays a crucial role in the in vitro and in vivo survival of both the parasite [149] and its host red cell [150]. For RBCs and reticulocytes, maintaining the cellular redox balance is vital as an imbalanced production of reactive oxygen species (ROS) as a consequence of a malfunction in the redox system may lead to oxidative stress, lipid peroxidation and decreased osmotic resistance [151], thus resulting in the cell death of haemopoietic precursors [152] and eryptosis or lysis of circulating red cells (erythrocyte apoptosis). In turn, oxidative stress-induced eryptosis and haemolysis are detrimental to parasite survival. *Plasmodium* parasites also have an own redox system, which has been shown to be vital for their survival [153]. Less is known of the *P. vivax* redox metabolism when compared to *P. falciparum* [154]; thus, it is possible that *P. vivax* may be more sensitive to oxidative stress. Also, little is known of the possible interplay between the parasites’ and host red cell redox systems [155, 156], which may be important in the maintenance of a positive redox balance that allows for mutual maturation in the case of *P. vivax* and the reticulocytes. In vitro, the ability to maintain the intraerythrocytic redox balance while properly supporting the *Plasmodial* redox metabolism may be dependent on the supply of the right (micro)nutrients in appropriate concentrations through the medium. Some micronutrients (such as copper, zinc, manganese [157], selenium [158]) are known to play an important role in the ability of reticulocytes and RBCs to prevent excessive oxidative stress, while others are pro-oxidant and dangerous for the integrity of red cells [159]. It is possible that the anti- or pro-oxidant activity of these micronutrients may be concentration dependent and that such elements may exert different effects on the *Plasmodium* than on the host red cell. In this context, the addition of specific antioxidants could be attempted that have been shown to have positive effects on the redox balance of the red cells and may potentiate the redox equilibrium in the *P. vivax* in vitro blood stage culture.

### Lipids: key players in need of tight regulation

*Plasmodium* spp. sharply grow and multiply during the trophozoite and schizont stages of their blood cycles. Active lipid metabolism accompanies the membrane proliferation associated with growth and merozoite production. Thus, malaria parasites are forced to meet their high demand for lipids through networks of both synthesis and scavenging [160]. A dynamic lipid balance is installed between the parasite, the host cell (including RBC, reticulocyte and hepatocyte) and the environment (plasma), which is crucial for parasite survival and has consequences for RBC early senescence and reticulocytes maturation as the parasite actively modifies its environment leading e.g. to increases RBC membrane and fragility [115]. In this context, it has recently been established that susceptibility to malaria infection as well as the parasite’s intrinsic virulence and growth rate are modulated by the host’s nutritional status, which can be sensed by the parasite [161, 162]. It is possible that suboptimal culture conditions e.g. in terms of medium nutrients may be sensed by the malaria parasite triggering a plethora of adaptation mechanisms that cannot necessarily be sustained long-term. The medium composition is therefore crucial to the establishments of long-term *P. vivax* in vitro blood stage cultures. In the context of lipids, a number of parasite peculiarities needs to be taken into account when formulating the medium for *P. vivax*: the parasite uses a unique enzymatic pathway for the synthesis of isopentenyl diphosphate [163, 164]; the parasite uses the bifunctional enzyme, FPP synthase, to synthetize isoprenoids; sphingolipids are obtained both by direct synthesis and scavenging, whereby an excess in ceramides appears to be toxic for the parasite through depletion of glutathione; malaria parasites possess a FAS II pathway for the synthesis of fatty acids, the activity of which during blood stages is modulated by the nutritional status of the host or the composition of the medium [161, 162]. Overall, the absence of FASII activity in *Plasmodium* blood stages under normal conditions suggests that precursors for desaturation can also be salvaged from the host. While a number of pathways for the synthesis of phospholipids have been identified in many *Plasmodium* species, substrates for the de novo synthesis of phospholipids need to be acquired from the environment. The incorporation of exogenous fatty acids [165–167] and lyso-PC [168] is necessary to sustain phospholipid synthesis and parasite growth during the intraerythrocytic stage and *P. falciparum* is able to take up ethanolamine [169], choline [170] and myo-inositol, from the host erythrocyte. Serine is likely imported via amino acid transporters or obtained from the catabolism of haemoglobin in the food vacuole.

While a selective PC transport mechanism also exists, which provides PC from the host cell directly to the parasite [171]. When formulating the medium it is important to consider that the parasite is incapable of de novo synthetizing cholesterol, for which it normally establishes a host supply through two potential sources: from circulating high-density lipoprotein particles (HDL particles) or directly from the erythrocytic membrane [160]. HDL molecules have been reported to be essential for malaria parasite schizogony [172] and for the delivery of cholesterol to the plasma membrane of the mature erythrocyte [173]. Freshly prepared human high-density lipoprotein fractions (concentration range of 0.25 to 0.50 mg/mL) have been used to support growth of *P. falciparum*, with results comparable to those obtained using human serum [174]. Other lipoprotein fractions, low- and very-low-density lipoproteins, produced little or no growth. Lipoproteins are important not only for their role in the parasite’s cholesterol supply, but also for their role in the remodelling of the reticulocyte. Both *P. vivax* and the reticulocyte are maturing entities and that their maturation needs to take place side by side as the reticulocyte ensures the healthy environment needed for the parasite’s growth and egress [175]. Thus, it needs to be considered that the study of reticulocyte remodelling during maturation is in its infancy and that complete maturation to a biconcave disc, has not been described to date to occur in vitro [176]. Reticulocytes loose approximately 20% plasma membrane and undergo the continued removal of residual intracellular components during their maturation. Moreover, results obtained with other *Plasmodium* spp. indicate that the parasites are capable of modulating the membrane lipid composition of host cells. In the case of host erythrocytes, it was found that *P. falciparum* infection led to an altered phospholipid molecular species composition, with a shift in the fatty acyl chain composition towards that of the parasite [177].

The most recent proposed model of reticulocyte maturation postulates a continuum of differential remodelling of the lipid bilayer and of the membrane-skeleton that leads to the mature circulating RBC. Within this process, at first, the selection at the plasma membrane for endocytosis takes place followed by a sorting step on the membrane of the sorting endosome. In this model the maturation of reticulocytes to RBCs is driven by a membrane raft-based mechanism for the sorting of disposable membrane proteins to exosomes [177]. As the decrease in membrane surface area that occurs in the reticulocyte-to-RBC maturation is of the same order of magnitude as the observed “increase” in band 3 and GPC, the latter can only be explained by the increase in concentration that follows a selective loss of cholesterol (and other components different from band 3) by the reticulocyte in the process. Therefore, it can be speculated that the addition of lipids, lipid precursors and cholesterol to the medium has to be tightly regulated in order e.g. to prevent a stiffening of the reticulocyte membrane, which may have consequences for parasite egress at the end of schizogony and in order to ensure the normal maturation of the reticulocyte. Furthermore, it has been suggested that the spleen may play an essential role in promoting some of the molecular changes in the reticulocyte during maturation [177] and that the maturation of healthy reticulocytes will not happen in absence of the spleen which may have serious consequences for the maturation and egress of *P. vivax* parasites in vitro. Overall, it is clear that more studies into the interplay between *P. vivax* and the reticulocyte are needed to make rational choices when it comes to the addition of lipids, lipid precursors and cholesterol to the medium. While it is clear that such components are likely to be essential for *P. vivax* maturation, their effects on the natural and parasite-driven reticulocyte remodelling should also be considered. In this context, it has to be acknowledged that more research is needed as we know relatively little on the interplay between parasite factors and reticulocyte factors when it comes to their impact on reticulocyte and parasite maturation mechanisms.

### Serum: an essential player

Serum is of paramount importance as it represents an extra source of nutrients. When dialyzed, human serum loses its ability to support growth of parasites due to the elimination of low molecular weight and soluble compounds [178]. Commercially available serum and serum acquired from healthy donors are nowadays considered reliable sources, which are both rendered devoid of complement by exposing them to 57 °C for 30 min. Whether this heat-inactivation also destroys some nutrients that are essential to parasite maturation or to the healthy maturation of the reticulocytes is unknown. Serum concentration has varied considerably between attempts (ranging from 10 to 50%). In *P. vivax* blood-stage cultures, the serum may be of particular importance as a balanced source of micronutrients for the parasites, but it also contains elements that are essential for the healthy maturation of the young reticulocyte populations [179–182] that *P. vivax* prefers to invade. For reasons that include cost, reproducibility, and possible presence of inhibitory immune factors and anti-malarial drugs, there was interest in substituting other types of mammalian sera (bovine, monkey, horse, goat, sheep, rabbit, or swine) for human serum or even developing a serum-free medium for in vitro blood-stage parasite culture. In comparing horse, swine, and lamb sera, horse serum was superior to the others but not as good as human serum [146]. Fetal bovine serum was found to be generally less effective in promoting *Plasmodium* growth than human serum [147]. Although freshly isolated fetal bovine serum of different cattle breeds initially produced good parasite growth, long-term continuous parasite growth was hampered as shown by a decline in parasite numbers over 30 days [183]. Rabbit serum (5 to 10%) was used in place of human serum but required a 2- to 3-week culture adaptation period [184].

By comparing *P. falciparum* growth in sera of different animal origins, Jensen found that all sera tested other than human serum are suboptimal in supporting *P. falciparum* in vitro expansion [185]. Ifediba and Vanderberg [186] reported replacement of human serum by Proteose Peptone no. 3 as well as by neopeptone, which was also tested by Divo et al. [177]; bovine serum albumin (5 g/L) with Cohn fraction IV was used to replace serum by Ofulla et al. [187]. McCoy’s 5A contains bacto-peptone. Commercial samples of freshly frozen or lyophilized human serum supported *P. falciparum* growth at about one-quarter the rate of freshly isolated human serum. Heat-inactivated, semi-immune human plasma from a region of malaria endemicity has been used successfully in the continuous cultivation of primary isolates of *P. falciparum* [187]. Pooling sera minimizes variations in growth-promoting properties of serum samples obtained from different humans [183] and rabbits [184]. There is also increased interest in the use of commercial serum replacements. Lingnau et al. [73] used a commercially available serum replacement preparation, Nutridoma-SR (4%), to support the growth of several strains of *P. falciparum* from different global locations, achieving a parasitaemia of about 10% within 3 to 4 days. Flores et al. [189] had better results using a lower concentration of Nutridoma-SR (1%) combined with Albumax I (0.5%), a purified serum albumin preparation. Cultures were maintained for 30 to 50 days, at about 10% parasitaemia compared to 15% parasitaemia achieved with human serum. They found that cultures raised in higher concentrations of Nutridoma-SR (2 or 4%) in combination with Albumax I were non-viable or gave lower levels of parasitaemia. Binh et al. [190] also used Albumax I in *P. falciparum* blood-stage cultures and obtained parasitaemias as high as 85% after 7 days with continuously passaged *Plasmodia*. Cranmer et al. using Albumax II (0.5%) in the presence of hypoxanthine achieved parasitaemias of about 6 and 12% for two different *P. falciparum* strains. Non heat-treatment plasma has been used for large-scale *P. falciparum* [191] culture; clotting was avoided by using plastic culture vessels or siliconized glassware. Growth-promoting factor GF 21 (containing an ammonium sulfate fraction of adult bovine serum plus insulin, transferrin, and sodium selenite) has also been tested as part of Daigo’s T basal medium for serum-free growth of *P. falciparum* [81] and *P. lophurae* [192]. Furthermore, RPMI 1640 was supplemented with adenosine, unsaturated C fatty acids, and fatty acid-free bovine serum albumin for serum-free growth, but growth rates of parasites were lower than those in plasma-containing medium [127]. 750 mg/L of cholesterol lipid concentrates have also been utilized [61] in this context. As previously detailed, attempts have been made at growing different *Plasmodium* spp. in medium enriched with RBC and liver extracts. In some cases, this was shown to be beneficial likely due to the fact that it provides the parasites with a reservoir of (micro)nutrients such as amino-levulinic acid, which have been shown to be essential in all stages of the parasite’s life cycle including the mosquito and liver stages [193]. However, clumping of erythrocytes in the presence of RBC extracts was also observed [194–196] which suggests that the benefits of adding extracts to support the long-term in vitro blood stage culture of *P. vivax* should be systematically evaluated.

## Conclusion

The last decade saw a paradigm shift in the fight against malaria from malaria control to malaria eradication. While the incidence of *P. falciparum* has declined since this paradigm shift took hold, it has become clear that for eradication to be achieved, *P. vivax*—the second major human malaria parasite—characterized by intrinsic peculiarities in its biology needs to be tackled. Thus, the urgent need to put *P. vivax* research back on the agenda. The major hurdle in the development of appropriate tools (e.g. gene disruption technologies) to study the complex biology of this malaria parasite species lies in the impossibility to culture *P. vivax* in vitro.

**Fig. 4:**
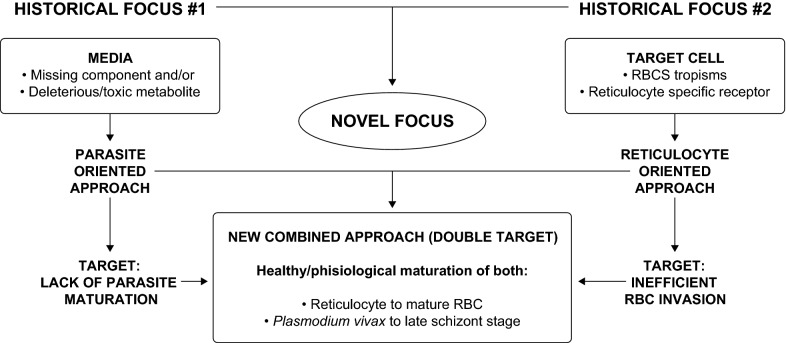
Progression of a healthy, short-term (5 days) *P. vivax* in vitro culture: T0 h rings, T20 h and T42 h (corresponding to a first cycle of in vitro cultivation; (D) T68 h, and T92 h corresponding to a second cycle of in vitro cultivation); and T116 h (corresponding to a third cycle of in vitro cultivation)

Parasite in vitro growth is a combination of three subsequent step: invasion, maturation and egress. Recent reviews have considered at length aspects of invasion processes, but neglected the other aspects. Based on recent discoveries on the role of nutrient sensing in *Plasmodium’s* pathophysiology in this review article we challenge the wide-held belief that it is sufficient to find the right parasite isolate and give it the right type of cells to invade for *P. vivax* to grow in vitro and put forward the view that culture conditions and the media in particular play an essential role in *P. vivax* adaptation to in vitro growth (Box 1). In this article, a critical review on the extensive body of literature concerning *Plasmodium* culture conditions is performed, with a specific focus on new and old culture media used in attempts to culture different *Plasmodium* spp. and related parasites. An analysis of the media compositions in detail and a dissection of the likely impact of the different components on the parasite’s in vitro fitness is carried out, while also looking at the likely effect on the maturation of the parasite’s host cell, the reticulocyte. In this context, the novel concept that the healthy maturation of both the parasite and its host cell, the reticulocyte, is necessary for a successful in vitro adaptation of *P. vivax* parasites is put forward (Fig. 4). Last, alternatives to current media practices that could contribute to a healthy development of *P. vivax* and its host cell in parallel, ultimately leading to merozoites fully capable of egressing, invading other reticulocytes and achieving continuous exponential growth between cycles are suggested and avenues for further research are proposed.

**Box 1:**
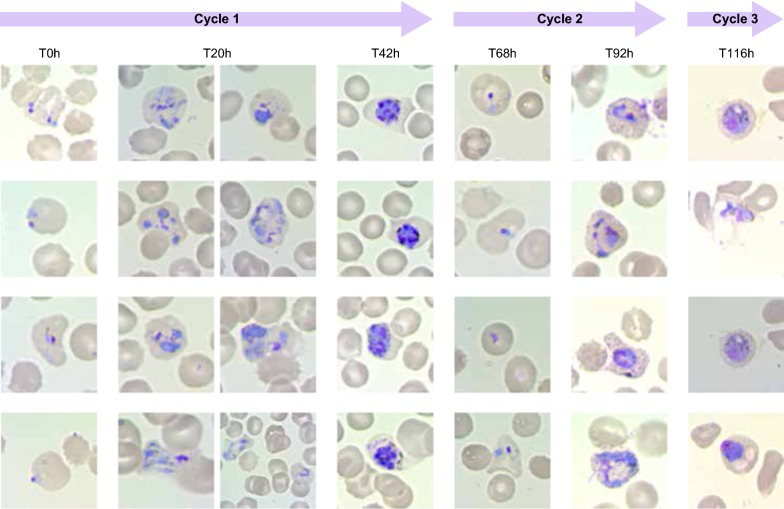
Summary of the proposed paradigm shift necessary to allow *Plasmodium vivax* long-term in vitro growth. Diagram explaining the necessary combination of two historically distinct focused approaches and postulating the need for a physiological maturation of both, the parasites and its host cell (reticulocyte) side-by-side to achieve a continuous, long-term in vitro *P. vivax* blood stage culture

## Supplementary information

**Additional file 1: Table S1.** Comparison of the components of alternative media used in attempts to culture *P. vivax* in vitro. Media composition of RPMI-1640, Waymouth and McCoy5A, L15, IMDM and Williams from Sigma. William’s medium is commercially available with or without glutamine, phenol red, sodium bicarbonate and Glutamax.

## Data Availability

Not applicable.

## Abbreviations

WHO: World Health Organization
RBC: red blood cell
PB: peripheral blood
HSC: haematopoietic stem cell
RT: room temperature
RPMI: Roswell Park Memorial Institute
BGM: Modified Harvard Medium
BME: Eagle’s Basal Medium
MEM: Eagle′s Minimum Essential Medium
DMEM: Dulbecco′s Modified Eagle′s Medium
GMEM: Glasgow’s Medium
OBM: Osteoblast Medium
HPGM: Haematopoietic Growth Medium
ACF: animal component-free
IMDM: Iscove’s Modified Dulbecco’s Medium
Mn^2+^: manganese
pABA: 4-aminobenzoic acid
CoA: co-enzyme A
ROS: reactive oxygen species
HDL: high‐density lipoprotein
GF 21: growth-promoting factor
